# Examination on level of scale efficiency in public hospitals in Tanzania

**DOI:** 10.1186/s12962-021-00305-4

**Published:** 2021-08-11

**Authors:** Fatuma Fumbwe, Robert Lihawa, Felician Andrew, George Kinyanjui, Eliaza Mkuna

**Affiliations:** 1 Faculty of Social Sciences, Department of Economics, Mzumbe University, P.O Box 5, Morogoro, Tanzania; 2 Senior Researcher, University of Cape Town, Cape town, South Africa

**Keywords:** Scale efficiency, Public hospitals, Data envelopment analysis

## Abstract

**Purpose:**

Tanzania has implemented policies that aim at improving health sector performance as well as the general health status of citizens. Establishment of community insurance fund, increase government budget allocation in health sector, establishment of institutions for critical and special diseases like Tanzania Ocean road cancer institute, Muhimbili Orthopaedic Institute and many other that aim at improving sector efficiency. These efforts and policies had a direct impact on improving the health sector and achieving Sustainable Development Goals (SDGs). Despite these improvement efforts, the health sector continues to face enormous challenges. Among the major challenges identified is the level of inefficiencies in healthcare delivery. It is for this reason; this paper examines the scale efficiency level in Tanzania’s public hospitals.

**Methods:**

Using data from the Ministry of Health, this paper employs the Input based Data Envelopment Analysis (DEA) to examine Tanzania’s public hospital efficiency levels. DEA has been applied because it can handle multiple inputs and output that can have different units simultaneously.

**Results:**

Findings showed that the average scale efficiency was 78.6%.and 72.9%for regional and district hospitals respectively. Additionally, 43.8% of the regional referral hospitals attained the most productive scale size compared to 21.05% in district hospitals.

**Conclusion:**

The study concludes that there is dire need for the ministry of health to consider resource reallocation across public hospitals. Periodic re-estimation of efficiency levels coupled with increased health care input injection is of urgent need.

## Background

The estimation of efficiency in Tanzania’s public hospitals is crucial in evaluating health policy initiatives and making comparative health analyses [2]. Improvement in the efficiency of hospitals can lead to a decrease in the government spending on healthcare services. Moreover, the saved funds can be reallocated in the expansion of other healthcare programs like rehabilitation, cure and increase engagement in diseases’ preventive programs. In the end this will have great impacts in the development of the quality of the provided health services in a country [17].

In improving the efficiency in the health sector, the Government of Tanzania has increased efforts to address health sector challenges. Among others, the efforts include construction of health facilities throughout the country, training health experts, increasing the budget for medication, implementation of various Health Sector Strategic Plans (HSSP I, II, III and the current HSSP IV of 2015 to 2020) and enhance country medical store department (MSD).In spite of the efforts, it has been observed that efficiency in the health sector has continued to be a significant problem. A case in point, in 2018, there were 350 health centers and 1000 dispensaries countrywide, whereby one medical doctor was attending approximately 26,000 to 30,000 patients per year [17]. This ratio is higher than the requirements of the international standards based on the WHO recommendation for developing countries, which advised that one medical doctor should attend a maximum number of 10,000 patients per year. On the other hand, the WHO [16] reported that, Tanzania has 0.39 nurses and 0.26–0.30 clinical staff (medical doctors, assistant medical officers and clinical officers) per 1000 population. This indicates that, on average, there is one prescriber (generally mid-level providers trained in-country, rather than medical doctors) in each primary facility with the workload averaging 29 outpatients per clinician per day in health centers and 20 in dispensaries [12]. Regarding hospital facilities the World Bank [15] reported that hospital beds for the Tanzania were 0.7 units per thousand people in 2010 which is 50% short from 1976 ratio of 1.4 units per thousand people. It is for this reason the WHO had recently ranked Tanzania healthcare system at 156 out of 191 countries with the overall efficiency index of 0.422 [17].

Inefficiency in the public health sector has further being caused by persistent government under funding in the sector. For instance, between the years 2002 and 2013, in real terms, the government of Tanzania’s health expenditure as a percentage of total health expenditure decreased from 45% to 36%. High proportions of Tanzania’s total health expenditure are financed by foreign donors that reached to 48% in fiscal year 2011/12 with the household/out-of-pocket spending of 33% in 2011/12 rather than from sustainable or prepaid sources such as general government revenue or health insurance [14]. In addition, Tanzania’s current Health Sector Strategic Plan IV that has reached the midterm of its implementation, realized government revenue to fund the plan, and other social-sector objectives did not meet initial projections. Substantial resource gaps remain to finance the full implementation of the plan, estimated at 21,945 billion Tanzanian shillings (TZS) for the period of 2015–2020 [9]. While mobilizing and pooling other sources of funds for the health sector remains difficult, fear remains that it might result into continuous delays in translating the country’s National Health Financing Strategy into policy.

Despite the fact that the WHO [17] has shown there is a problem of efficiency in the Tanzania’s health sector, it is still unclear about the precise level of efficiency (both technical and scale in public hospitals) in the country. Thus, this paper examines the scale efficiency level in Tanzania’s public hospitals using Data envelopment analysis, for 2016 secondary data from public hospitals in Tanzania. Scale efficiency has been used in this study because it can be applied at the point that the production share of each health facility (hospitals) is optimal when they produce at minimum average costs. This paper will contribute to giving information on improving Tanzania public hospitals’ efficiency. Also, it adds valuable information of the efficiency in public hospitals that will be used by all important and interested stakeholders who will help in public hospitals’ management auditing and other necessary procedures for the development of the health sectors in a country.

## Theoretical perspective of data envelopment analysis (DEA)

The term efficiency explains the degree of performances which portrays the lowest amount of inputs used in the creation of the highest amount of outputs. Therefore, it is quantifiable, measurable and analyzed by the use of ratios of output to the available total input.

$$Efficiency = outputs/inputs$$

A number of techniques can be used to estimate efficiency. The study has applied DEA which explains managerial capability to choose the optimum size of the hospital, as hospital size may result into having efficiency or inefficiency.

DEA is a non-parametric technique of estimating efficiency by using linear programming method. It does not need an explicit functional form and can construct the frontier from the observed inputs and outputs ratios. Charness et al. 4 formulated a DEA model which is mathematically expressed as:

$${\text{Max}}_{{{\theta ,}}} {\uplambda }^{{\uptheta }}$$

Subject to

$$\begin{array}{*{20}{c}} & X\lambda \le x_{0} \\ & \theta \le Y \lambda \\ & \lambda \ge 0 \end{array}$$

whereas X is the output of $i$ input of a matrix that has ${x}_{i}$ number of columns, another output $Y$ from the matrix of ${y}_{i}$ number of columns with $\uplambda$ being an input of vector $i X 1$. By the use of DEA, producers’ performance problems are determined through their abilities in expanding the output vector subjected to their constraints that have been imposed with the available practices that yield optimal outputs. If producers’ radial grow this possible, then the optimal $\theta$ will be greater than one and equal to one when growth is not going to be reached at all.

DEA as a non-parametric approach has various benefits which help in making it becoming more influential and better in terms of theoretical perspective [10]. DEA can be categorized into constant return to scale and variable return to scale and it can fall under two models or approaches of input and output models. The input model represents the inefficient unit, which is made to be efficient by proportionate reduction on the input, while the proportion of the output is held constant. The output is expanding in the output model by taking input into control [13]. Moreover, DEA has been an appropriate measure for the estimation of efficiency in hospitals, since the price data is hard to obtain and the multi-output productions are relevant.

Many authors (scholars) prefer the application of DEA methods due to several advantages like simultaneous use of multiple inputs and outputs, it does not require a mathematical specification of the production function, it is most appropriate to investigate the impact of exogenous variables, suggests recommendations for an inefficient production unit. On the other hand, its application spectrum is eliminated by several disadvantages or limitations. The most important are results are sensitive to outlier values; it’s just about measuring relative efficiency, limitation for the sample size. When choosing a DEA model, it is necessary to define initially if the input or output-oriented method will be used. The used methods are different for application to the healthcare sector.

### Empirical literature review

Number of literatures has applied DEA in examining the efficiency of various private and public institutions. Most of these literatures have chosen DEA based on the linear programming ability of the DEA in producing feasible choices. Moreover, in measuring scale efficiency and there are two types of scales that are Constant Return to Scale (CRS) and Variable Return to Scale (VRS). CRS assumes that an increment in inputs results in proportion increment in outputs. This implies that there is no significant relationship between the size of DMU and efficiency. Meanwhile, VRS assumes that an increment in inputs results in a disproportionate increment in outputs [1, 5].

Recent literature have assessed different aspects of efficiency in hospitals such as Dong and Li [11] examined efficiency in the Chinese public hospitals in 2012. They used Bootstrap DEA methods in analyzing technical efficiency. To avoid double-counting problems or to mix allocative and technical efficiency in the selection of output/input indicators, the study used quantity of available beds and hospital staff as input indicators while numbers of diagnosed patients and discharged inpatients were regarded as output indicators. The study revealed that there were 8 hospitals with an efficiency score of 1 using the traditional BCC model. The new score of the efficiency revealed that 5 hospitals had excellent performance, 1 hospital had an average performance, and 5 hospitals had an average performance with ample scope for improvement. Furthermore, 2 hospitals needed to improve performance and 1 hospital needed urgent improvement. Despite the strength of Bootstrap DEA used, the study had some failures as all environmental factors were considered as a random factor in the estimation of efficiency score. Moreover, the study could estimate efficiency by DEA’s and SFA during first and second stage respectively and traditional BCC-DEA model in the third stage to re-estimate efficiency [11].

Hamidi11 measured the efficiency of government hospitals in Palestine using stochastic frontier approach (SFA). The study’s objectives were to estimate the technical efficiency and measures the effects of numbers of bed and health sector technical and non-technical human resources. The study collected data from the ministry of health, for the period of 6 years from 2006 to 2012 for 132 observations. The author used numbers of doctors, nurses, beds as well as nonmedical staff as inputs. Nevertheless, the number of admitted patients, the average length of stay, number of days being hospitalized, operations cases as well as the number of outpatients visited the hospital were regarded as outputs variable. Furthermore, the study explored that the mean technical efficiency reached 55%, and medical doctors and nurses are important factors in the hospital production.

Flokou et al.9 applied DEA in measuring the efficiency of a public hospital in Greece from2009 to 2013 following the financial crisis of 2007–2008. Specifically, their study intended examining the efficiency of government-owned health facilities from 107 Greek hospitals. The study used inputs such as the number of beds in a hospital, medical professionals, while the in-patients, and outpatients, were used as output variables. The study used two years DEA in assessing the scale and technical efficiency. Moreover, the study analyzed the basis of production changes between two consecutive years by using the Malmquist productivity index. The study found some improvement in the scale and technical efficiencies at the end of five year of the financial crisis period. However, the study did not use a parametric approach in the estimation of efficiency which is more effective in measuring productive efficiency of decision-making units. This study addressed this gap through the use of DEA to the estimation of efficiency in Tanzania

## Methodology

DEA can handle the efficiency estimates of multiple inputs and outputs without any judgment of relative importance of the difference inputs or outputs; this study adopted input-model as the public hospital have control over the inputs used in the production process than the output obtained. Moreover, the inputs orientation score signifies the maximum allowed extensive reduction of input capable for generating the same level of outputs. The study also adopted assumptions of return to scale under the input orientation since variables returns to scale is regarded as suitable in measuring hospital efficiency since public hospitals differ by size, different factor inputs like number of beds etc. Therefore, from the variables’ return to scale, the problem of linear programming is explained by:

Minimize θ

Subject to

$$- y_{jm} + \sum\limits_{j = 1}^{M} y_{jm} \,\lambda_{j} \, \ge \,0,m = \,1,2.$$

$$\theta x_{jk} - \,\sum\limits_{j = 1}^{m} x_{jk} \,\lambda_{j} \, \ge \,0,\,k = 1.$$

$$\sum\limits_{j = 1}^{M} {y_{j} = 1}$$

$${\uplambda }_{{\text{j}}} \, > \,0,\,{\text{j}}\,{ = }\,{1,}\,{2}.....{\text{m}}$$

### Definition and measurement of variables

The study obtained secondary data from 19 regional referral hospitals and 119 districts hospitals collected in 2016. Choice of these hospitals is because they provide service to a great number of populations, and they are the biggest consumer of health sector resources. Furthermore, considering inputs and outputs that were selected for the study, these hospitals were appropriate. Therefore, estimation of efficiency for these hospitals can be generalized for all public hospitals in Tanzania mainland (see Table 1).

**Table 1:** Variables of the study. Source: Own calculation, 2018

	Variables	Description
Input	Beds	Includes number of beds available in each health facility
	Medical staff	Includes number of medical doctors, nurses, clinical officers, and medical attendant
Output	Inpatients	Includes number of inpatients or admission recorded by the facility in 2016
	Outpatients	Includes total number of outpatients visited the facility in 2016

## Results of the study

### Scale efficiency of district hospitals

24 out of 114 district hospitals which is equivalent to 21.05% had efficiency score of 1, which show scale efficiency of 100%. The remained 90 district hospitals 78.95% of the total district hospitals were scaled inefficiently. 25 hospitals out of 90 inefficient district hospitals had a scale efficiency of less than 0.5. 24 hospitals had a scale efficiency of between 0.51 and 0.70 and the remained 41 hospitals had an efficiency score of between 0.71 and 0.99. Within 90 inefficient district hospitals, 87.8% (79 hospitals) of hospitals were found to have increasing return to scale (IRS), and 12.2% (11hospitals) of hospitals had decreasing return to scale (DRS). This finding shows that 87.8% of the inefficient district hospitals in Tanzania for the year 2016 were small and needed to expand their scale of operation while the 12.2% of the inefficient district hospitals needed to offset some of excess inputs in operation in order to attain constant return to scale (CRS) (see Table 2).

**Table 2:** Summary of scale efficiency for district hospitals. Source: Researcher’s own calculation

Scale efficiency score	Frequency	Percent	Cumulative percentage
0.00–0.50	25	21.93	21.93
0.51–0.70	24	21.05	42.98
0.71–0.99	41	35.96	78.95
1	24	21.05	100.00
Total	114	100.00	

### Scale efficiency of regional referral hospitals

As shown in Table 3, out of 16 studied regional referral hospitals the average scale efficiency was 0.786. In addition, 7 out of 16 regional hospitals equivalent to 43.75% attained 100% efficiency as they had a scale efficiency score of 1. These 7 regional referral hospitals attained the most productive scale size (MPSS), and hence they obtained constant return to scale (CRS). The remaining 9 hospitals equivalent to 56.25% were scale inefficient regional referral hospitals as they obtained efficiency score of less than 1. Moreover, 2 out of these 16 regional hospitals equivalent to 22.2% of all regional hospitals had efficiency score of less than 0.50, 3 hospitals equivalent to 33.3% of the hospitals had efficiency score of between 0.51 and 0.70 and 4 (44.4%) hospitals obtained efficiency score of between 0.71 and 0.99. However, all 9 scale inefficient regional referral hospitals had an increasing return to scale (IRS) meaning that the inefficient hospitals are too small for their operation to operate at their MPSS thus they need to expand their scale of operation so as for them to attain the CRS.

**Table 3:** Results from DEAP efficiency summary. Source: Researcher’s own calculation

Hospitals ID	Scale efficiency	Return to scale type
1	1	CRS
2	0.781	IRS
3	1	CRS
4	0.774	IRS
5	0.633	IRS
6	0.267	IRS
7	1	CRS
8	0.858	IRS
9	0.28	IRS
10	0.882	IRS
11	1	CRS
12	1	CRS
13	1	CRS
14	0.576	IRS
15	1	CRS
16	0.527	IRS
Mean	0.786	

### Amount of inputs available for reallocation and output increase potentials in district hospitals

Slacks show resources underuse or overuse at the hospital, and hence enables suggestion on whether there should be an adjustment on inputs used to attain efficiency. Since in the analysis of study input orientation was assumed, increase or decrease of inputs has to be done to achieve hospital efficiency. For the district hospitals, 39.5% of hospitals do not require inputs reallocation and the remained 60.5% hospitals can reallocate its inputs to achieve efficiency. District hospitals could reallocate its inputs by transferring from efficient hospitals to inefficient ones.

Slacks in the outputs shows that inputs are underutilized, thus reallocation of the inputs might achieve efficiency. Moreover for hospitals with outputs slacks and no inputs slacks, measures such as more knowledge on the importance of using hospitals. Since some Tanzanian still prefer the use of traditional treatment than going to hospital, these measure can increase the inpatient and outpatients of hospitals. If district hospitals were working as a group were required on average to increase inpatients by 736 and outpatients by 15517 without changing the quantity of inputs. Also they were required to decrease inputs such as beds by 2, medical doctor by 3, Nurse by 5, clinical officer by 1 and medical attendant by 11 to attain efficiency.

### Amount of inputs available for reallocation and output increase potential in regional referral hospitals

With the presented slacks showed in Table 4, 62.5% (10) regional referral hospitals do not require any adjustment and remained 37.5% need reallocation to achieve efficiency. To achieve efficiency, regional referral hospitals as a group could on averagely increase output by 1131 in inpatients and 5160 in outpatients without changing inputs mix. As well as averagely decrease its’ inputs by 3 in medical doctors, 7 nurses, 1 clinical officer, and 5 medical attendants.

**Table 4:** Summary of input and output slacks for regional referral hospital. Source: Researcher’s own calculation

Hospitals	Beds	Medical doctors	Nurse	Clinical officer	Medical attend	Inpatient	Outpatient
1	0.000	0.000	0.000	0.000	0.000	0.000	0.000
2	0.000	0.000	34.711	2.545	22.969	0.000	16779.768
3	0.000	0.000	0.000	0.000	0.000	0.000	0.000
4	0.000	6.793	24.643	0.121	31.551	0.000	23087.879
5	0.000	6.793	24.643	0.121	0.000	5482.162	0.000
6	0.000	0.000	0.000	0.000	0.000	0.000	0.000
7	0.000	0.000	0.000	0.000	0.000	0.000	0.000
8	0.000	30.977	10.553	7.497	30.132	2622.338	0.000
9	0.000	8.078	15.252	0.000	0.000	7716.948	36736.866
10	0.000	0.000	0.000	0.000	0.000	0.000	0.000
11	0.000	0.000	0.000	0.000	0.000	0.000	0.000
12	0.000	0.000	0.000	0.000	0.000	0.000	0.000
13	0.000	0.000	0.000	0.000	0.000	0.000	0.000
14	0.000	0.000	0.000	0.000	0.000	0.000	0.000
15	0.000	0.000	0.000	0.000	0.000	0.000	0.000
16	0.000	0.192	4.236	0.000	0.000	2277.168	5947.797
Average	0.000	3.302	7.127	0.643	5.291	1131.164	5159.519

## Discussion of findings on scale efficiency in public hospitals

The average scale efficiency for regional hospitals was 78.6%. 43.8% of the regional referral hospitals attained the most productive scale size, and 56.25% were scale inefficient with increasing return to scale. Increase return to scale means enjoying economies of scale thus increase in one input use results to more increase in output. This implies that the inefficient hospitals are too small for their operation to operate at their MPSS thus they need to expand their scale of operation so as for them to attain the CRS. When compared, these findings are consistent to Bwana [3] who found that overall efficiency for Volunteering Agency Hospitals in Tanzania by 2012 had no improvement with the scale efficiency at 55.08%.

The average scale efficiency was 72.9% for district hospitals. 21.05% of the district hospitals attained the most productive scale size, termed as scale efficient hospital and the remained 78.95% did not attain MPSS and termed as scale inefficient district hospitals. For scale inefficient hospitals, 87.8% had increasing return to scale and the remaining 12.25% had decreasing return to scale. However, Harrison and Sexton [8] study in US showed that there were improvements in number of religious hospitals between 1998 and 2001 from 72% to 74%. Hospitals with IRS are too small for their operation and are needed to expand scale of operation, while those with DRS are needed to scale down their operation.

### Limitations of DEA

The hospitals used in the study are not the actual presentation of all public hospitals in the country. whereas majority of these hospitals are found in urban areas they vary a lot in terms of medical personnel grades and qualifications. Highly trained medical personnel are likely to be found in hospitals located in urban places. DEA is limited in controlling for these hospital level fixed effects. Therefore, the results may not be generalizable to peripheral health units particularly those found in the rural areas.

Secondly, despite of the strength and quality of the DEA it does not provide a way for the absolute efficiency measures. The biggest advantage of using DEA approach in this study is its ability to accommodate simultaneously measurement in the production of hospital care goods (outputs) without ignoring poor outcomes that detract from overall social welfare. One of the major limitations is that the productivity of the hospitals in our sample are all assessed by a best practice frontier whose definition is limited by including all hospitals in our sample. In essence, the study gauged hospital efficiency in a relative rather than an absolute sense. Because of this limitation, it is not likely to make any definitive statements about absolute quality. However, it is noted that the relative reduction in congestion is necessary albeit not sufficient condition in maximizing patient and hospital welfare.

Finally, as in most other hospital studies, the study faced limitations in the detail of data available. Most of the data elements, such as medical doctors, are only available at the hospital not service line of patient care unit level. Unfortunately, in this analysis, the study was limited to beds as an indicator of capital input. However, hospitals with a larger number of beds also typically invest more in other types of capital such as imaging equipment or hemodialysis machines. Future research may be able to resolve some of these limitations as better risk-adjustment methods are developed in the future and as additional data become available. As more data become available, refinements such as how different ownership in different states is related to congestion, may be worthy of investigation. Certainly, replication of our approach using data from more recent year and with additional subsets of quality indicators will be worthwhile to assess whether our principal findings are robust.

## Policy recommendations

The Tanzania Ministry of Health, Ministry of Finance and Planning and other concerned ministries need to increase efforts on reducing inefficiency in public hospitals. Based on the findings that there is a high level of inefficiency in district hospitals and some regional referral hospitals were inefficient in 2016. The finding revealed that for the year 2016, 56.25% and 78.95% of regional referral and district hospital respectively were scaled inefficient. Regular estimation of efficiency both technical and scale efficiency in public hospitals must be done in order to ease decision making processes and improving health sector resource allocation.

Ministry of Health, Community Development, Gender, Elderly and Children (MoHCDGEC), President’s Office Regional Administration and Local Government (PORALG) can help in the timely available of funds (budgets) to procure (secure) hire sufficient inputs resources such as medical doctor, nurses, medical attendant, clinical officers and beds to enable public hospitals attain efficiency. Since study revealed that some hospitals have serious underutilization problems as there are excess inputs which is not equivalent to the output produced which create inefficiency and therefore reallocation is prerequisite and unavoidable. Moreover, hospitals with shortage resources need to be provided with sufficient resources to attain efficiency, and hospitals with excess needs to be considered in reallocation of the inputs.

Also other stakeholders and government can provide education on importance of getting medical help from hospitals curative, treatment and preventive assistance. Increase of this knowledge can increase the need for hospitals service and thus utilize the resources allocated to the hospitals, hence increase efficiency. Moreover, increasing level of efficiency in public hospitals depict improvement in the provision of health service and availability of better health services, thus attaining Tanzania vision of 2025 and Sustainable Development goals 2030.

On the other hand, district and regional hospital administrators have to adopt these strategies to facilitate enhancing inputs which will help in efficiency improvement and loss minimization. However, number of limitations in this study such as the decision on the use of variables has been based on literatures read and authors’ discussions. Therefore, given these challenges policy makers should take into considerations and use more evidence from other studies to make decisions.

## Data Availability

Data will be available upon request.

## Appendix

See Table 5.

**Table 5:** Summarization of inputs and outputs—district hospitals

Hospital	Bed	MD DCT	Nurse	CLNOFF	MEDATT	IPD	OPD
1	0.0	8.6	6.8	0.0	0.0	400.4	14993.5
2	0.0	3.4	0.0	0.0	13.2	0.0	0.0
3	0.0	0.7	0.0	0.0	8.2	0.0	0.0
4	0.0	0.0	0.0	0.0	0.0	0.0	0.0
5	32.2	0.0	8.7	0.0	32.8	1542.4	1284.7
6	0.0	4.7	0.0	0.0	30.2	2397.8	26120.5
7	0.0	1.2	0.0	0.0	0.0	2680.2	35235.0
8	0.0	0.0	0.0	0.0	0.0	0.0	0.0
9	0.0	0.0	11.7	0.0	54.2	1797.2	31523.4
10	0.0	0.4	0.0	0.0	2.8	0.0	24021.2
11	0.0	2.6	4.7	0.0	0.0	0.0	12808.6
12	0.0	4.9	0.0	0.0	0.0	916.4	1253.8
13	0.0	0.0	37.4	0.0	3.2	0.0	25964.2
14	0.0	8.8	0.0	0.0	6.4	0.0	0.0
15	0.0	0.0	0.0	0.0	0.0	0.0	0.0
16	0.0	3.4	0.0	0.0	1.0	3670.8	0.0
17	0.0	3.1	6.0	0.0	0.0	0.0	28028.5
18	0.0	0.0	0.0	0.0	0.0	0.0	0.0
19	0.0	1.5	0.8	0.0	0.0	3171.7	33365.2
20	0.0	10.4	8.5	0.0	0.0	3052.0	0.0
21	0.0	1.3	1.5	0.0	0.0	0.0	24975.5
22	0.0	1.2	0.0	0.0	0.0	0.0	28043.5
23	0.0	2.0	0.0	0.0	22.2	0.0	11764.3
24	0.0	2.0	2.1	0.0	0.0	3714.5	44287.1
25	0.0	4.7	0.0	0.0	2.1	0.0	6388.3
26	0.0	0.0	0.0	0.0	0.0	0.0	0.0
27	18.8	0.0	21.8	0.0	61.8	3404.1	555.0
28	0.0	0.0	0.0	0.0	0.0	0.0	0.0
29	0.0	0.0	0.0	0.0	0.0	0.0	0.0
30	0.0	0.0	0.0	0.0	0.0	0.0	0.0
31	0.0	2.4	0.0	19.5	20.7	0.0	0.0
32	0.0	0.0	0.0	0.0	0.0	0.0	0.0
33	0.0	1.6	4.7	0.0	0.0	3300.3	33215.5
34	0.0	0.0	0.0	0.0	0.0	0.0	0.0
35	0.0	0.0	0.0	0.0	0.0	0.0	0.0
36	0.0	1.2	0.0	0.0	6.6	0.0	0.0
37	0.0	6.0	0.0	0.0	0.0	1192.0	19369.1
38	0.0	0.7	0.0	0.0	0.0	1634.7	27596.0
39	0.0	9.1	18.1	0.0	64.5	0.0	25339.3
40	0.0	0.0	0.0	0.0	1.0	296.5	18432.8
41	0.0	10.0	4.7	0.0	0.0	3540.9	11170.1
42	0.0	4.7	2.6	0.0	0.0	1062.7	23170.0
43	0.0	0.0	0.0	0.0	0.0	0.0	0.0
44	0.0	0.0	0.0	0.0	0.0	0.0	0.0
45	0.0	0.0	0.0	0.0	17.0	0.0	2413.0
46	0.0	4.7	0.0	0.0	0.0	0.0	20250.7
47	0.0	2.5	5.6	0.0	0.0	0.0	25699.2
48	0.0	0.0	0.0	0.0	0.0	0.0	0.0
49	0.0	4.7	3.0	0.0	0.0	3317.0	40554.3
50	0.0	0.0	13.3	0.0	80.7	3930.7	15076.5
51	0.0	0.0	13.3	0.0	70.8	2897.5	17289.1
52	0.0	7.4	3.2	0.0	0.0	601.3	22519.0
53	0.0	0.0	0.0	0.0	0.0	0.0	0.0
54	0.0	0.4	0.0	0.0	0.0	1929.8	5092.3
55	0.0	0.3	10.8	0.0	0.0	1228.4	17347.3
56	0.0	0.0	0.0	0.7	47.3	0.0	63639.8
57	0.0	0.0	0.0	0.0	7.4	0.0	28492.0
58	0.0	4.5	0.6	0.0	0.0	982.8	18372.9
59	0.0	0.0	0.0	0.0	0.0	0.0	0.0
60	0.0	0.7	0.0	0.0	0.0	0.0	0.0
61	0.0	0.0	0.0	0.0	0.0	0.0	0.0
62	0.0	0.0	28.8	0.0	0.0	0.0	67901.3
63	0.0	55.0	265.1	0.0	335.0	0.0	2929.4
64	0.0	10.4	0.0	0.0	21.6	5262.0	18372.7
65	0.0	0.0	0.0	1.6	0.0	0.0	29637.8
66	70.0	2.0	0.0	0.0	0.0	2689.0	15660.0
67	0.0	2.9	0.0	0.0	0.0	3987.7	32358.7
68	0.0	3.5	4.6	0.0	0.0	0.0	44565.3
69	0.0	0.0	0.0	0.0	0.0	0.0	0.0
70	0.0	0.0	0.0	0.0	0.0	0.0	0.0
71	0.0	7.6	0.0	0.0	17.5	0.0	1211.2
72	0.0	0.0	0.0	18.2	1.0	0.0	63179.1
73	0.0	0.0	0.0	0.0	0.0	0.0	0.0
74	0.0	0.0	0.0	1.7	38.1	0.0	33050.5
75	0.0	0.0	0.0	1.0	75.0	0.0	10014.5
76	0.0	0.0	0.0	0.0	0.0	0.0	0.0
77	0.0	0.0	56.2	0.0	0.0	45.1	33904.7
78	0.0	0.0	0.0	0.0	0.0	0.0	0.0
79	0.0	3.1	0.0	0.0	3.1	3708.9	31659.1
80	0.0	2.6	0.0	0.0	0.0	0.0	21754.5
81	0.0	2.8	0.0	0.0	4.8	170.1	58.5
82	0.0	0.0	0.0	0.0	0.0	0.0	0.0
83	0.0	0.0	0.0	0.0	9.1	0.0	0.0
84	0.0	0.0	0.0	0.0	3.4	0.0	0.0
85	0.0	0.0	3.0	0.0	0.0	2157.1	36718.5
86	0.0	2.0	2.3	0.0	0.0	0.0	21941.0
87	0.0	9.3	0.0	6.0	0.0	2845.7	22188.5
88	0.0	0.0	0.0	0.0	0.0	0.0	0.0
89	62.5	0.8	18.5	0.0	0.0	0.0	211455.6
90	0.0	3.5	0.0	0.0	1.1	0.0	85894.8
91	0.0	0.0	0.0	0.0	0.0	0.0	0.0
92	0.0	2.8	3.3	0.0	0.0	4105.3	42023.3
93	0.0	1.1	0.0	0.0	0.0	19.7	15614.9
94	0.0	9.6	0.0	0.0	61.8	0.0	0.0
95	0.0	5.1	0.0	0.0	4.6	0.0	0.0
96	0.0	0.0	0.0	0.0	0.0	0.0	0.0
97	0.0	0.0	0.0	0.0	0.0	0.0	0.0
98	0.0	1.0	0.0	0.0	0.0	80.8	7054.7
99	0.0	2.4	0.0	0.0	0.0	318.6	15534.4
100	0.0	5.1	0.0	0.0	11.1	1293.5	18692.0
101	0.0	0.0	0.0	0.0	0.0	0.0	0.0
102	0.0	3.6	0.0	0.0	2.2	585.7	31753.6
103	0.0	0.0	0.0	0.0	0.0	0.0	0.0
104	0.0	0.6	0.0	0.0	0.0	0.0	84.4
105	0.0	0.0	0.0	0.0	0.0	0.0	0.0
106	0.0	0.0	0.0	0.0	0.0	0.0	0.0
107	0.0	0.0	0.0	0.0	0.0	0.0	0.0
108	0.0	0.0	0.0	0.0	0.0	0.0	0.0
109	0.0	11.5	0.0	0.0	46.0	0.0	0.0
110	0.0	0.0	6.9	0.0	32.1	1041.6	26476.5
111	0.0	3.6	0.0	0.0	23.4	1778.5	0.0
112	0.0	10.0	0.0	0.0	22.4	878.6	16983.2
113	0.0	0.0	0.0	0.0	0.0	0.0	0.0
114	0.0	0.0	0.0	9.9	0.0	245.4	24626.4
Average	1.610	2.526	5.075	0.514	11.118	735.750	15517.108
